# Prognostic Impact of 9-Month High-Sensitivity C-Reactive Protein Levels on Long-Term Clinical Outcomes and In-Stent Restenosis in Patients at 9 Months after Drug-Eluting Stent Implantation

**DOI:** 10.1371/journal.pone.0138512

**Published:** 2015-09-25

**Authors:** I-Chang Hsieh, Chun-Chi Chen, Ming-Jer Hsieh, Chia-Hung Yang, Dong-Yi Chen, Shang-Hung Chang, Chao-Yung Wang, Cheng-Hung Lee, Ming-Lung Tsai

**Affiliations:** Department of Cardiology, Percutaneous Coronary Intervention Center, Chang Gung Memorial Hospital, Chang Gung University College of Medicine, Linkou, Taoyuan, Taiwan; University of Bologna, ITALY

## Abstract

**Introduction:**

The level of 9-month high-sensitivity C-reactive protein (hsCRP) in predicting cardiovascular outcomes is scanty in patients at 9 months after receiving drug-eluting stent (DES) implantations. This study aims to evaluate the relationship between 9-month follow-up hsCRP levels and long-term clinical outcomes in patients at 9 months after receiving DES.

**Methods:**

A total of 1,763 patients who received 9-month follow-up angiography were enrolled and grouped according to hsCRP level 9 months after the DES implantation: group I (718 patients, hsCRP<1.0 mg/L), group II (639 patients, 1.0≦hsCRP≦3.0 mg/L), and group III (406 patients, hsCRP>3.0 mg/L).

**Results:**

Group III patients had a lower cardiovascular event-free survival rate than group I or II patients during a follow-up of 64±45 months (64.5% vs. 71.6% vs. 72.8%, respectively, p = 0.012). Multivariate analysis showed that a follow-up hsCRP level <3.0 mg/L was an independent predictor of a major adverse cardiovascular event (cardiac death, reinfarction, target lesion revascularization, stenting in a new lesion, or coronary bypass surgery). Group III patients had a higher restenosis rate (11.3% vs. 5.8% vs. 6.6%, respectively, p = 0.002) and loss index (0.21±0.32 vs. 0.16±0.24 vs. 0.18±0.28, respectively, p = 0.001) than group I or II patients in 9-month follow-up angiography.

**Conclusions:**

A high 9-month follow-up hsCRP level is an independent predictor of long-term clinical cardiovascular outcomes in patients at 9 months after DES implantation. It is also associated with a higher restenosis rate, larger late loss and loss index at 9 months after DES implantation.

## Introduction

Coronary artery disease (CAD) is the major cause of death globally. Inflammation plays a central role in the pathogenesis of atherosclerosis as well as plaque instability [1]. C-reactive protein (CRP), an acute phase reactant, is recognized as an important marker of vascular wall inflammation and as a strong predictor of future cardiovascular events [2, 3].

Percutaneous coronary intervention (PCI), and especially stent implantation, stimulates arterial intimal cellular proliferation and extracellular matrix synthesis which is mediated largely by inflammatory processes [4]. Several studies have shown an association between pre-procedural CRP levels and subsequent cardiac events in patients treated with bare metal stent (BMS) implantations [5–9]. However, other studies have reported that using CRP level to predict cardiovascular outcomes in patients receiving mixed BMS and drug-eluting stent (DES) or only DES implantations is controversial [10, 11]. In addition, the clinical follow-up duration in the majority of related studies is short (around 1 year). Furthermore, the prognostic value of CRP level in in-stent restenosis (ISR) is inconclusive [12–14]. Patients at 9 months after PCI should be considered as “relative” stable CAD patients (or are very close to such patients). It is known that CRP is primarily synthesized and secreted rapidly in the liver 4–6 hours after an acute inflammatory stimulus [15], after which the level returns to its baseline level. Theoretically, the CRP level after the acute phase is more stable and can reflect the actual atherosclerotic status, and may therefore be a more reliable predictor of long-term cardiovascular outcomes in these patients. A previous study also showed that the 180-day concentration of high-sensitivity (hs) CRP was strongly associated with the progression of baseline moderately obstructive lesions in non-culprit vessels that required coronary angioplasty [16]. The aim of this study was to evaluate the relationship between 9-month follow-up hsCRP levels and long-term clinical outcomes in patients undergoing PCI using DES only. The relationship between hsCRP level and angiographic outcomes after 9 months of follow-up was also evaluated.

## Materials and Methods

### Patient population and laboratory analysis

The Cardiovascular Atherosclerosis and Percutaneous TrAnsluminal INterventions (CAPTAIN) registry is a prospective, physician-initiated, single-center observational database which includes the data of 6,300 patients who underwent elective or emergent PCI with stenting at our hospital between November 1995 and December 2013. Ethical approval of this study was obtained from the Institutional Review Board of Chang Gung Medical Foundation. All the patients provided informed consent to undergo the procedure and follow-up protocol including long-term assessment and agreement of publication prospectively.

The inclusion criteria for stenting were: evidence of myocardial ischemia and >50% stenosis in a native coronary artery or in a bypass vein graft that was suitable for stenting. The exclusion criteria were: severe multi-vessel disease requiring bypass surgery, contraindication for aspirin or clopidogrel use, and refusing to undergo the procedure. We enrolled 1,763 consecutive patients from the CAPTAIN registry who underwent DES implantation between July 2003 and June 2013,all of whom received 9-month angiographic follow-up (75% follow-up rate of total patients received DES implantations). None of the patients had concurrent inflammatory conditions such as infection, inflammatory arthritis and connective tissue disease, malignancies, or had undergone recent (<2 months) surgery or suffered major trauma. Blood samples for hsCRP were collected before the stenting procedure and at the 9-month angiographic follow-up. The hsCRP level was measured on a Hitachi LST 008 automatic analyzer (Tokyo, Japan) using an immunoturbidimetric assay. Dual antiplatelet therapy with aspirin and ticlopidine /clopidogrel was administered to all of the patients for at least 9 months. Patients with acute coronary syndrome had higher CRP levels than those with stable angina pectoris. As the enrolled patients in this study are close to stable CAD patients, therefore, they were divided into 3 groups according to the 9-month follow-up hsCRP level of <1, 1 to 3, and >3mg/L.

### Interventional procedure and clinical follow-up

The stent implantation was performed through the femoral or radial artery according to standard techniques. A predilation was performed using an undersized balloon if the lesion was very tight (>70% stenosis). The type of stent implanted was decided by the operator, mainly on the basis of the available stent size. After initial stent deployment, high-pressure balloon inflation (≧14 atm) was applied. Cardiac isoenzymes were measured in all of the patients immediately and 6 hours after the procedure. A total of 2,562 stents were implanted into 2,401 lesions, including 749 Cypher (Johnson & Johnson, Warren, New Jersey, USA), 760 TAXUS (Boston Scientific, Natick, Massachusetts, USA), 189 Endeavor (Medtronic, Minneapolis, MD, USA), 365 Resolute (Medtronic, Minneapolis, MD, USA), 268 Xience (Abbott Vascular, Santa Clara, California, USA), 91 Biomatrix (Biosensor, Singapore), 105 Promus (Boston Scientific, Natick, Massachusetts, USA), and 35 Nobori (TERUMO, Tokyo, Japan). Information on clinical status, medical management, and occurrence of any adverse events was obtained from the patients’ medical records. The patients were clinically followed up through outpatient visits or telephone contact. Clinical follow-up visits were scheduled at 1, 2, 3, 6, 9 and 12 months, and every 3 months thereafter. Angiographic follow-up was recommended after 9 months or earlier in cases of suspected recurrent myocardial ischemia.

### Angiographic analysis

Quantitative angiographic analysis was conducted with a selected end-diastolic cine-frame that showed stenosis in its most severe and non-foreshortened view. A contrast-filled guiding catheter was used as reference for calibration. Random measurements were performed by two blinded, experienced angiographers. The inter observer correlation coefficient (r) was 0.93 (p<0.01), and the intra observer correlation coefficient was 0.95 (p<0.01). The minimal luminal diameter (MLD), reference vessel diameter, percentage of diameter stenosis, and balloon diameter were measured by the automatic edge-detection method. Binary restenosis was defined as diameter stenosis of ≧50% occurring in the segment inside the stent or 5 mm segment proximal or distal to the stent at follow-up angiography (in-segment restenosis). Acute gain was defined as the difference between the baseline and the final MLD, late loss as the difference between the final post-stenting and follow-up MLD, net gain as the difference between acute gain and late loss, and loss index as the ratio of late loss to acute gain. The left ventricular ejection fraction was measured from the left ventricular angiogram obtained at a right anterior oblique projection with an angle of 30°.

### Definitions

We defined a major adverse cardiovascular event during the follow-up period as cardiac death, reinfarction (ST-segment elevation or non-ST-segment elevation myocardial infarction), target lesion revascularization (TLR), stenting in a new lesion, or necessitation of coronary bypass surgery. ST-segment elevation myocardial infarction was diagnosed by standard methods if the patient experienced prolonged chest pain (longer than 30 minutes) that could not be relieved by nitroglycerin, showed an ST-segment elevation of ≧0.2 mV in at least two contiguous electrocardiographic leads, and had significantly elevated creatine kinase-MB enzyme levels.

### Statistical analysis

We used STATA statistical software (version 10) for all statistical analyses. The final results were represented as mean±standard deviation or as percentages, and categorical data were presented as numbers. The normality of all of the variables was analyzed. Continuous variables were compared in the three groups with one way analysis of variance (ANOVA). To assess determinants of dependent variables, we estimated logistic odds ratios (ORs) with their 95% confidence intervals (CIs). Multivariate Cox proportional hazard regression analysis with the forward stepwise selection process was used to determine independent predictors of a major adverse cardiovascular event. Event-free survival was analyzed using the Kaplan-Meier method. A p value less than 0.05 was considered significant.

## Results

### Baseline characteristics and hsCRP

A total of 1,763 patients with 2,401 lesions who underwent 2,562 DES implantations were enrolled in this study. The patients were grouped according to 9-month follow-up hsCRP level after the DES implantation: group I (718 patients, hsCRP<1.0 mg/L), group II (639 patients, 1.0≦hsCRP≦3.0 mg/L) and group III (406 patients, hsCRP>3.0 mg/L). With increasing tertiles of hsCRP level, more patients were older, women, hypertensive, had left ventricular dysfunction, cardiogenic shock on presentation, multi-vessel involvement, and less use of statin. In addition, baseline hsCRP levels were significantly higher with increasing tertiles of follow-up hsCRP levels, and the 9-month follow-up hsCRP level was lower than the baseline level in all three groups (Table 1).

**Table 1:** Patient characteristics by follow up hsCRP level.

	Group I (n = 718)	Group II (n = 639)	Group III (n = 406)	P value
Risk factors and history				
Age (years)	59.9 ± 11.3	61.2 ± 11.8	63.1± 11.1	< 0.001
Woman, n (%)	93 (13.0)	116 (18.2)	96 (23.6)	< 0.001
Diabetes mellitus, n (%)	204 (28.4)	179 (28.0)	139 (34.2)	0.066
Hypertension, n (%)	385 (53.6)	364 (57.0)	260 (64.0)	0.003
Current smoking, n (%)	286 (39.8)	252 (39.4)	153 (37.7)	0.768
Family history, n (%)	12 (1.7)	12 (1.9)	3 (0.7)	0.318
Hyperlipidemia, n (%)	353 (49.2)	326 (51.0)	215 (53.1)	0.458
Previous stroke, n (%)	28 (3.9)	29 (4.5)	28 (6.9)	0.072
Initial clinical presentation				
ACS, n (%)	315 (43.9)	269 (42.1)	172 (42.4)	0.782
STEMI, n (%)	209 (29.1)	176 (27.5)	95 (23.4)	0.115
NSTEMI-ACS, n (%)	106 (14.8)	93 (14.6)	77 (19.0)	0.111
LVEF < 40%, n (%)	67 (9.3)	68 (10.6)	57 (14.0)	0.049
Cardiogenic shock, n (%)	21 (2.9)	20 (3.1)	23 (5.3)	0.043
Multi-vessel disease, n (%)	411 (57.2)	389 (60.9)	264 (65.0)	0.035
Medications				
Statin, n (%)	609 (84.9)	517 (80.9)	304 (75.1)	<0.001
Antiplatelet, n (%)	676 (94.3)	605 (94.7)	374 (92.3)	0.276
β-blocker, n (%)	655 (91.4)	572 (89.5)	363 (89.7)	0.458
Baseline LDL level, mg/dl	105.3±38.1	105.9±79.4	101.7±35.0	0.481
hsCRP level, mg/L				
Baseline	11.3 ± 27.4	15.0 ± 29.4	20.2 ± 37.3	< 0.001
Follow up	0.6 ± 0.2	1.8 ± 0.5	13.6 ± 26.7	< 0.001

ACS: acute coronary syndrome;

hsCRP: high-sensitivity C-reactive protein;

LVEF: left ventricular ejection fraction;

NSTEMI: non-ST-segment elevation myocardial infarction;

STEMI: ST-segment elevation myocardial infarction.

Table 2 shows the characteristics of the lesion in the three groups. There were 964 lesions in group I, 885 in group II, and 552 in group III. Higher hsCRP levels were noted in the patients with more complex lesions (p = 0.033).

**Table 2:** Lesion characteristics by follow-up hsCRP level.

	Group I (n = 964)		Group II (n = 885)		Group III (n = 552)		P value
Type							0.033
Type A, n (%)	9	(0.9)	2	(0.2)	6	(1.1)	
Type B1, n (%)	162	(16.8)	121	(13.7)	82	(14.9)	
Type B2, n (%)	430	(44.6)	426	(48.1)	229	(41.5)	
Type C, n (%)	363	(37.7)	336	(38.0)	235	(42.6)	
Target vessel location							< 0.0001
Left main, n (%)	27	(2.8)	22	(2.5)	13	(2.4)	
LAD, n (%)	454	(47.1)	395	(44.6)	270	(48.9)	
LCx, n (%)	201	(20.9)	157	(17.7)	89	(16.1)	
RCA, n (%)	253	(26.2)	273	(30.8)	154	(27.9)	
Others, n (%)	29	(3.0)	36	(4.4)	26	(4.7)	
Lesion morphology							
Segmental, n (%)	525	(54.5)	499	(56.4)	297	(53.8)	0.838
Eccentric, n (%)	336	(34.9)	346	(39.1)	186	(33.7)	0.069
Calcified, n (%)	127	(13.2)	131	(14.8)	81	(14.7)	0.548
Angulation, n (%)	28	(2.9)	23	(2.6)	14	(2.5)	0.768
Thrombosis, n (%)	146	(15.1)	106	(12.0)	48	(8.7)	0.008

LAD: left anterior descending artery;

LCx: left circumflex artery;

LIMA: left internal mammary artery;

OM: obtuse margin;

PDA: posterior descending artery;

PL: postero lateral;

RCA: right coronary artery.

### Angiographic analysis

Follow-up angiography was performed in 718 patients with 964 lesions in group I 287±60 days after stenting, 639 patients with 885 lesions in group II 287±58 days after stenting, and 406 patients with 552 lesions in group III 276±67 days after stenting (Table 3). Before stenting, there were no differences in the diameters of stenosis or MLD among the three groups (p = 0.906, and p = 0.851, respectively). Group I patients had the largest reference vessel diameter among the three groups (p = 0.002). After stenting, group I patients had the largest MLD among the three groups (3.00±0.44, 2.96±0.43, and 2.92±0.40 mm, respectively, p = 0.002). After 9 months of follow-up, group III patients had the largest diameter of stenosis (24% vs. 19% vs. 20%, respectively, p<0.001) and a smaller MLD (2.41±0.83 vs. 2.63±0.69 and 2.55±0.71 mm, respectively, p<0.001) than the patients in groups I and II. Group III patients also had a larger late loss (0.50±0.77 vs. 0.38±0.58 vs. 0.41±0.60 mm, respectively, p = 0.001) and loss index (0.21±0.32 vs. 0.16±0.24 vs. 0.18±0.28, respectively, p = 0.001) than the patients in groups I and II. The restenosis rate was higher in group III patients than in groups I and II patients (11.3 vs. 5.8% vs. 6.6%, respectively, p = 0.002).

**Table 3:** Quantitative angiographic measurements.

	Group I	Group II	Group III	P value
Patients with 9-month follow-up	718	639	406	
Number of lesions	964	885	552	
Days to follow-up	287±60	287±58	276±67	
Before stenting				
% diameter stenosis	84±14	84±13	84±13	0.906
MLD (mm)	0.50±0.45	0.49±0.43	0.50±0.41	0.851
RVD (mm)	3.21±0.45	3.17±0.45	3.13±0.41	0.002
After stenting				
% diameter stenosis	7±6	6±7	7±5	0.729
MLD (mm)	3.00±0.44	2.96±0.43	2.92±0.40	0.002
RVD (mm)	3.21±0.45	3.17±0.44	3.13±0.41	0.001
Follow-up				
% diameter stenosis	19±17	20±19	24±24	<0.001
MLD (mm)	2.63±0.69	2.55±0.71	2.41±0.83	<0.001
RVD (mm)	3.22±0.43	3.17±0.43	3.14±0.39	0.002
Acute gain (mm)	2.50±0.57	2.47±0.56	2.42±0.51	0.018
Late loss (mm)	0.38±0.58	0.41±0.60	0.50±0.77	0.001
Net gain (mm)	2.12±0.77	2.05±0.78	1.96±1.22	0.004
Loss index	0.16±0.24	0.18±0.28	0.21±0.32	0.001
Restenosis rate	42/718 (5.8%)	42/639 (6.6%)	46/406 (11.3%)	0.002

MLD: minimal luminal diameter;

RVD: reference vessel diameter.

### Long-term clinical outcomes

**Fig 1:**
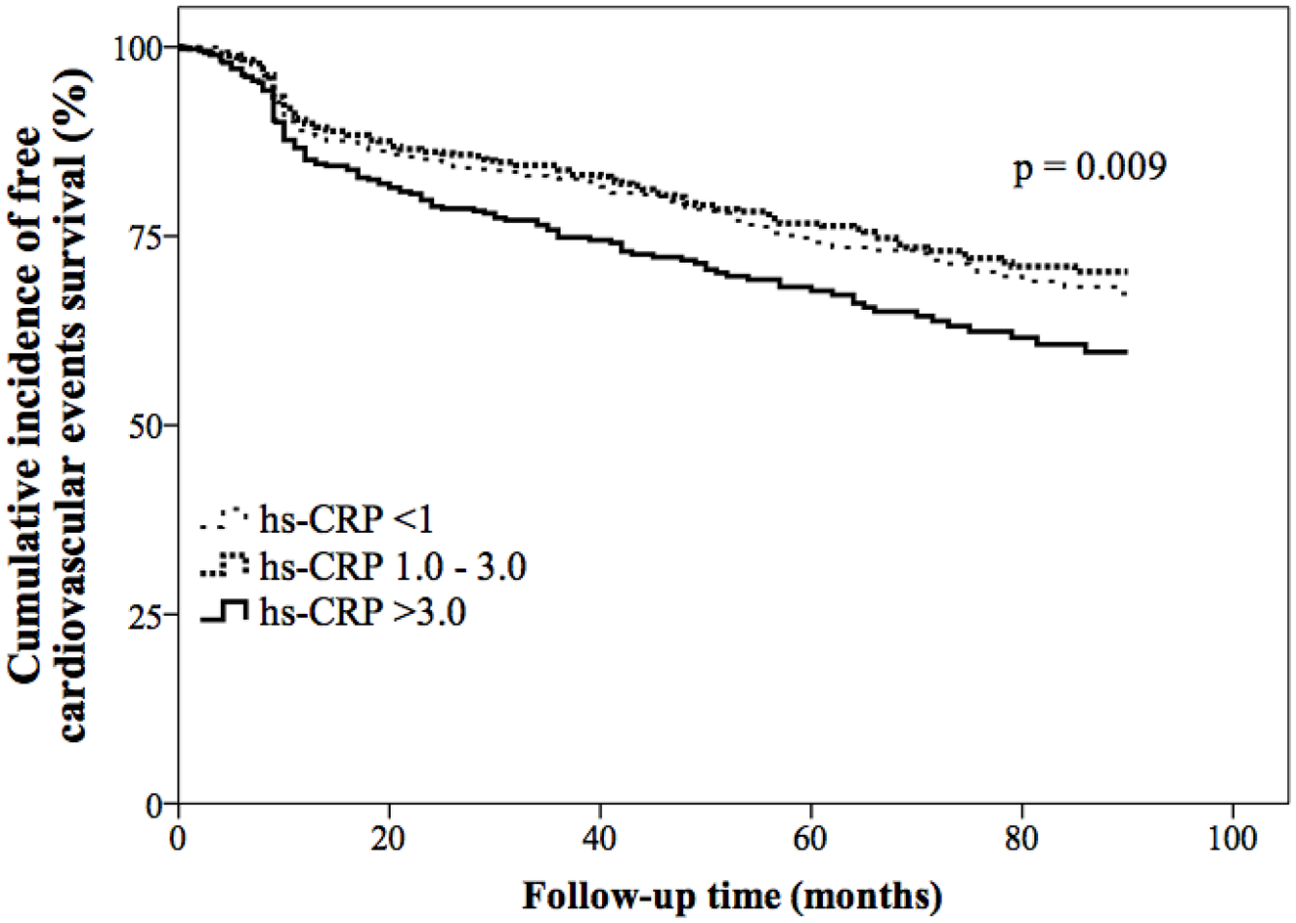
The Kaplan-Meier analysis showed different cardiovascular event-free survival rate across the three groups (p = 0.009).

Group III patients had a higher incidence of overall mortality than groups I and II patients (7.6% vs. 2.5% vs. 3.3%, respectively, p<0.001), as well as cardiac mortality (5.4% vs. 1.9% vs. 2.0%, respectively, p = 0.001), reinfarction (5.9% vs. 2.9% vs. 1.9%, respectively, p = 0.001), and TLR (10.1% vs. 5.2% vs. 5.0%, respectively, p = 0.001). There were no differences in the incidence of new lesions requiring stenting, coronary bypass surgery, and non-fatal stroke among the three groups during the follow-up period of 65±45 months. Group III patients also had a lower cardiovascular event-free survival rate than groups I and II patients (64.5% vs. 71.6% vs. 72.8%, respectively, p = 0.012) (Table 4). The cardiovascular event-free survival rate obtained by performing Kaplan-Meier analysis differed across the three groups (p = 0.009) (Fig 1). Univariate (Table 5) and multivariate (Table 6) predictors of cardiovascular events included diabetes mellitus (HR: 1.43; 95% CI: 1.17–1.74; p<0.001), multi-vessel coronary artery disease (HR: 2.41; 95% CI: 1.92–3.02, p<0.001), and a follow-up hsCRP level>3.0mg/L (HR: 1.29; 95% CI: 1.02–1.62, p = 0.035).

**Table 4:** Clinical events during follow-up.

	Group I		Group II		Group III		P value
Number of patients	718		639		406		
Mortality, n (%)	18	(2.5)	21	(3.3)	31	(7.6)	<0.001
Cardiac, n (%)	14	(1.9)	13	(2.0)	22	(5.4)	0.001
Non-cardiac, n (%)	4	(0.6)	8	(1.3)	9	(2.2)	0.047
Reinfarction, n (%)	21	(2.9)	12	(1.9)	24	(5.9)	0.001
Target lesion revascularization, n (%)	37	(5.2)	32	(5.0)	41	(10.1)	0.001
New lesion stenting, n (%)	105	(14.6)	68	(10.6)	55	(13.5)	0.085
Coronary bypass surgery, n (%)	6	(0.8)	8	(1.3)	3	(0.7)	0.640
Non-fatal stroke, n (%)	8	(1.1)	17	(2.7)	6	(1.5)	0.085
Cardiac event-free survival, n (%)	514	(71.6)	465	(72.8)	262	(64.5)	0.012

**Table 5:** Univariate analysis for predictors of major adverse cardiovascular events.

Predictors	HR	95% CI	P value
Age			0.915
< 55	1	-	-
55 to 75	1.05	0.84 ~ 1.31	0.657
>75	1.31	0.97 ~ 1.77	0.081
Woman	1.03	0.80 ~ 1.33	0.806
Diabetes mellitus	1.58	1.30 ~ 1.92	< 0.001
Hypertension	1.21	0.99 ~ 1.47	0.058
Current smoking	0.87	0.72 ~ 1.06	0.178
Family history	1.07	0.51 ~ 2.27	0.853
Hyperlipidemia	0.94	0.78 ~1.14	0.527
Previous stroke	1.41	0.93 ~ 2.15	0.108
STEMI on admission	0.84	0.68~1.05	0.127
NSTEMI-ACS on admission	1.03	0.89~1.34	0.843
LVEF < 40%	1.09	0.81 ~ 1.46	0.562
Cardiogenic shock on admission	1.31	0.80 ~ 2.12	0.281
Multi-vessel disease	2.52	2.02 ~ 3.16	< 0.001
Usage of statin	0.97	0.77~1.22	0.767
hsCRP, baseline			0.904
< 1.0	1	-	-
1.0 to 3.0	1.06	0.80 ~ 1.40	0.681
>3.0	1.02	0.79 ~ 1.30	0.901
hsCRP, follow up			0.002
< 1.0	1	-	-
1.0 to 3.0	0.91	0.73 ~ 1.15	0.429
>3.0	1.39	1.10 ~ 1.75	0.006

ACS: acute coronary syndrome;

hsCRP: high sensitivity C-reactive protein;

LVEF: left ventricular ejection fraction.

**Table 6:** Multivariate analysis for predictors of cardiovascular events.

Predictors	HR	95% CI	P value
Diabetes mellitus	1.43	1.17 ~ 1.74	< 0.001
Multi-vessel disease	2.41	1.92 ~ 3.02	< 0.001
hsCRP, follow up			0.009
< 1.0	1	-	-
1.0 to 3.0	0.89	0.70 ~ 1.11	0.296
>3.0	1.29	1.02 ~ 1.62	0.035

hsCRP: high-sensitivity C-reactive protein.

## Discussion

The major findings of this study are that a higher 9-month follow-up hsCRP level in patients at 9 months after PCI was: (1) associated with more adverse clinical and anatomic severity; (2) was an independent predictor of long-term clinical cardiovascular outcomes; and (3) was associated with a higher restenosis rate, larger late loss and loss index in angiographic follow-up.

### Patient characteristics and 9-month follow-up hsCRP

CRP, synthesized by hepatocytesin response to proinflammatory cytokines, is a circulating pentrax in involved in the human immune response, and one of the best-characterized inflammatory biomarkers. An elevated serum level of CRP is associated with an adverse prognosis in patients with stable or unstable angina and following acute myocardial infarction [2, 17, 18]. This elevated inflammatory marker rapidly declines after intracoronary stenting treatment when the acute insult subsides; however, the 180-day (the chronic phase) hsCRP level has been reported to be significantly higher in patients who require a second PCI than in patients who do not for the moderate obstruction of non-culprit vessels [16]. A previous study showed that pre-procedural hsCRP is associated to a large extent with the biological activity of coronary and cardiac disease processes rather than their anatomical severity. Furthermore, a history of recent acute myocardial infarction was independently associated with elevated hsCRP, whereas coronary lesion characteristics (multi-vessel disease, presence of peripheral or cerebral vascular disease, and prior revascularization) were not [11]. However, an elevated 9-month follow-up hsCRP level was associated with multi-vessel disease and complex coronary lesions, but it was not associated with the presentation of acute coronary syndrome in this study. This indicates that the hsCRP level after the acute phase is more stable and can reflect the actual residual atherosclerotic burden. In general, individuals who have high blood pressure, exercise less, are overweight, and smoke tend to have high levels of CRP. In this study, group III patients had more comorbidities (hypertension, multi-vessel CAD) and used less statin than groups I and II patients, which may have caused higher hsCRP levels. In addition, female gender was associated with an elevated 9-month follow-up hsCRP level in this study, which is consistent with a previous report [19]. The explanation for this observation is still unknown.

### Nine-month follow-up hsCRP and long-term outcomes after DES implantation

Pre-procedural CRP is most commonly used in the prediction of cardiovascular outcomes, however, the predictive role of pre-procedural CRP in clinical cardiovascular outcomes is controversial. Two previous studies enrolled patients with BMS and DES implantations [10, 11]. Palmerini et al. reported that the highest tertiles of pre-procedural CRP levels and acute coronary syndrome were associated with an increased risk of death / myocardial infarction at 9 months of follow-up after left main stenting, in which DES implantations were used in 51% of 83 patients [10]. However, a recently published study by Hermann et al. showed that a high hsCRP level was not an independent determinant of mortality after PCI in 513 patients with 79% of DES implantations during a follow-up period of 5 years [11]. Two other studies enrolled only patients who received DES implantations [20, 21]. Park et al. reported that the highest tertile of pre-procedural CRP levels was an independent predictor of major coronary events (cardiac death / Q-wave myocardial infarction) at 1year of follow-up in 1,650 patients [21]. Gaspardone et al. reported that the maximal change in CRP level (CRP at 48 hours / CRP at baseline) was significantly correlated with clinical outcomes at 12 months of follow-up. To the best of our knowledge, this study is the first to correlate 9-month follow-up hsCRP level with the longest clinical follow-up (65±45 months) in the largest patient cohort with DES implantations. Yip et al. reported that the hsCRP concentration was significantly higher in pre-procedural in patients with unstable angina undergoing coronary artery stenting than in the control group, and reported a marked decline after the procedure [16]. This indicates that hsCRP will return to its baseline concentration after an inflammatory stimulus has subsided. Therefore, the 9-month follow-up hsCRP level is more stable and can reflect the actual atherosclerotic status as well as long-term clinical outcome and restenosis rate at 9-month follow-up angiography in patients survived at 9 months after PCI. Dr. Kelly reported the PROSPECT study that enrolled 697 patients with acute coronary syndromes underwent PCI followed by gray-scale intravascular ultrasound (IVUS) and frequency IVUS. They found that untreated high-risk non-culprit lesions were more likely to cause subsequent MACE in patients with very elevated 6-month CRP levels (3 to 10 mg/L) when compared with normal levels (<3mg/L) [22]. This result is consistent with that of this study. It demonstrated that both high-risk plaques and systemic inflammation are necessary for development of late MACE. Inflammation may be an important driver of residual cardiovascular risk in patients with CAD. The CRP is thought to be associated with the “vulnerable patient”, a new concept that is comprised of vulnerable plaque [23], vulnerable myocardium (prone to ischemia), and vulnerable blood (prone to thrombosis) [24].

### Nine-month follow-up hsCRP and restenosis after DES implantation

The mechanism of ISR is a very complex pathophysiological process, and data regarding the prognostic value of CRP in ISR are conflicting. One meta-analysis which enrolled 1,062 patients from 9 prospective observational studies showed that elevated levels of pre-procedural CRP were associated with greater ISR after BMS implantation [12]. In contrast, other studies have reported no association between increased CRP levels and restenosis [13, 14]. The use of DES is associated with a lower restenosis rate than the use of BMS because of less neointimal hyperplasia. However, the clinical impact of a late inflammatory response on ISR after DES implantations is still under debate [21, 22], and data on the association between CRP levels after 6–9 months of follow-up and ISR after DES implantation are lacking. Xu et al. indicated that 8-month follow-up CRP levels could predict ISR in 303 patients with DES implantations [23], which is consistent with this study. In addition, Gottsauner-Wolf et al. found that increased CRP levels that persisted for longer than 96 hours were associated with a greater incident of ISR [24]. Our study showed that this late inflammatory response was related to the diameter of stenosis, late loss, loss index and ISR in angiographic follow-up after 9 months.

### Limitations

There are several limitations to this study. First, this is a prospective, observational study from a single center, and therefore the patient population and lesion characteristics may have been heterogeneous or biased. Second, all of the enrolled patients received follow-up angiography, which accounted for 75% of all CAPTAIN registry patients during the study period. It is possible that there was selection bias related to this incomplete angiographic follow-up. However, such bias is unlikely to be clinically relevant because this registry is an “all comer” registry. The other 25% patients refused to receive follow-up angiography because of doing well clinically. Third, we did not check serial changes of hsCRP during the entire follow-up period.

## Conclusions

A higher 9-month follow-up hsCRP level is associated with more adverse clinical and complex anatomic settings. It is also an independent predictor of long-term clinical cardiovascular outcomes in patients at 9 months after DES implantation. A higher follow-up hsCRP level was also associated with a higher restenosis rate, larger late loss and loss index in angiographic follow-up after 9 months in this study. Our results suggest that 9-month follow-up hsCRP level is a feasible predictor for long-term clinical outcomes and restenosis rate in patients at 9 months after DES implantations.
